# The Effect of High-Intensity Interval/Circuit Training on Cognitive Functioning and Quality of Life During Recovery From Substance Abuse Disorder. A Study Protocol

**DOI:** 10.3389/fpsyg.2019.02564

**Published:** 2019-11-15

**Authors:** Øyvind Andreassen, Kolbjørn Brønnick, Anne-Lill Njå, Einar Furulund, Sverre Nesvåg

**Affiliations:** ^1^Salvation Army Treatment Center Stavanger, Stavanger, Norway; ^2^Center for Alcohol and Drug Research, Stavanger University Hospital, Stavanger, Norway; ^3^Department of Public Health, Faculty of Health Sciences, Stavanger, Norway; ^4^Centre for Age-Related Medicine (SESAM), Stavanger University Hospital, Stavanger, Norway

**Keywords:** physical activity, high-intensity interval training, high-intensity circuit training, substance use disorder, brain health, neurocognition

## Abstract

This proposed study will examine whether structured physical activity reduces the recovery time of cognitive functioning during the early phase of substance use disorder treatment. Addiction or substance dependence is associated with neurobiological changes and cognitive impairment that can affect quality of life and the efficacy of therapy for up to a year after clinical detoxification. The biological, psychological, and social effects of physical exercise have the potential to be a therapeutic approach to increase quality of life and relieve symptoms associated with substance abuse, such as psychosis, depression, and anxiety. There is a dearth of research on physical activity and exercise in clinical substance use disorder patients. This protocol describes a clinical study that will examine cognitive recovery after substance abuse using physical exercise as a treatment intervention. We will use a quasi-experimental longitudinal clinical trial, with a pretest and multiple posttests, on naturally randomized sequential groups. Patients will be consecutively be recruited into the study groups, with a control group that is completed, before its followed by an intervention group, each with 30 patients. Patients will be enrolled 2 weeks after the start of detoxification, at which time all subjects will be inpatients at the Stavanger Salvation Army Treatment Center in the Norwegian specialized healthcare system. Cognition will be evaluated with a comprehensive battery of cognitive tests, including several tests of executive function. Physical fitness will be tested with the Rockport 1-Mile Walk Test, the 30-S Chair Stand Test, the 1-Min Burpee Test at baseline (within the first 2 weeks of admittance) and after 4 weeks. The intervention will be a 30-min workout at 70–90% of maximum heart rate (134–170 bpm), recorded and calculated by a Polar heart rate monitor. The intervention treatment will be administered four times a week for 4 weeks and will consist of high-intensity circuit training, high-intensity interval training, functional movement, and primitive reflex training. We anticipate improvement in both the control and intervention groups, with the exercise intervention group having the greatest increase in recovery of cognitive function because of the combination of functional full body movements and primitive movement training in an intense interval training program.

**Clinical Trial Registration ID:** ISRCTN74750479, Retrospectively Registered.

## Introduction

Substance use disorders (SUD) are among the most prevalent, chronic, and severe diseases in the world in terms of mortality and disability (Kessler et al., 2007), and they are associated with considerable social, economic, and individual health consequences (Adrian and Barry, 2003; Schuckit, 2006; Hasin et al., 2007; Kirby and Sugden, 2007; Wittchen et al., 2011). The combination of impaired physical and mental health among SUD populations is associated with life expectancies that are 20–30 years less than the general population (Stenbacka et al., 2010; Nordentoft et al., 2013). Individuals with SUD have a high tendency for comorbidity. Compared with the general population, the SUD group has more frequent contact with the healthcare system (Richards et al., 1999, 2017; Bernstein et al., 2014) because of a variety of illnesses such as cancer, diabetes, cardiovascular diseases, different kinds of physical trauma, and suicide (Kim et al., 2009; Stenbacka et al., 2010; Nordentoft et al., 2013). Depression, anxiety, attention deficit hyperactivity disorder (ADHD), personality disorder, and psychosis are among the most common comorbidities (Conway et al., 2006; Pennay et al., 2011; Pettinati et al., 2013; Adida et al., 2014; Harstad et al., 2014; Hartz et al., 2014). Individuals with SUD also have a high prevalence of cognitive impairment, which can contribute to low quality of life and the tendency to drop out of treatment (Rosselli and Ardila, 1996; Gouzoulis-Mayfrank and Daumann, 2006; Hagen et al., 2016, 2017a,b, 2019; Topiwala et al., 2017; Hall et al., 2018). A recent study (Hagen et al., 2016) also highlighted the importance of adequate recovery time for cognitive functions. Although pharmacological and psychological interventions are well established in the treatment and the research fields, relapse rates are typically high. The limited success rates of SUD treatments provide a good argument for focusing on adjunct treatments for SUD patients with interventions that target both physical and mental health.

Physical activity may be an excellent adjunct treatment to existing treatment regimens by improving the patient’s function and reducing symptoms. Exercise is a subcategory of physical activity that is planned, structured, repetitive, and designed to improve or maintain physical fitness, physical performance, or health (Caspersen et al., 1985). The general effects of physical activity are most significant when participants transition from an inactive lifestyle to a more physically active lifestyle (Pate et al., 1995; Ekelund et al., 2016). Exercise-based interventions are well established in the mental health service and research fields. Increased physical fitness, positive effects on depression and anxiety symptoms, and fewer negative symptoms for patients with schizophrenia are some of the results from mental health research (Rosenbaum et al., 2014; Haglund et al., 2015). Exercise interventions have also been observed to have positive effects on cognitive functions for severe mental illnesses (Firth et al., 2016), perhaps partially through an impact on brain functioning. Such research constitutes a persuasive argument for the exploration of exercise-based interventions in chronic or severe SUD populations.

However, only a few studies have examined the impact of physical activity or exercise in patients with SUD (Zschucke et al., 2012; Giesen et al., 2015). In September 2017, we conducted a literature search in Embase, Medline, SPORTDiscus, PsycINFO, and CINAHL using the search terms “high-intensity interval training” (HIIT), “high-intensity circuit training” (HICT), “cognitive function,” and “SUD.” The results of our search yielded 11 articles (Weinstock et al., 2008, 2014; Smith and Lynch, 2011; Wolff et al., 2011; Zschucke et al., 2012, 2013; Flemmen et al., 2014; Wang et al., 2014; Haglund et al., 2015; Klika and Jordan, 2015; Unhjem et al., 2016) of relevance to this study. This corpus included research that examined physical activity for SUD populations as outpatients or inpatients in recovery after withdrawal (Zschucke et al., 2012). Physical activity or exercise for SUD populations was shown to improve physical fitness, cardiovascular health, and sleep quality, and reduce the withdrawal symptoms of substance use, as well as anxiety and depression symptoms (Roessler, 2010; Zschucke et al., 2012; Wang et al., 2014; Giesen et al., 2015; Hallgren et al., 2017). It has also been observed that engaging in a physical activity program during treatment can contribute to reduced dropout rates, thereby increasing the success rate of therapy (Weinstock et al., 2014). Although this body of work presents promising results, many of the studies have methodological limitations, such as being pilot studies or lacking control groups. Because of the positive impact exercise can have on mental health, we believe it is extremely important to explore exercise interventions in the clinical treatment and recovery for SUD populations. Preexisting data and approaches in the field of mental health and SUD show great promise; for example, HIIT principles have been found to increase health benefits (Milanović et al., 2015; Unhjem et al., 2016; Karlsen et al., 2017). However, we lack data on dose, intensity, and the most beneficial types of exercise.

1. This study protocol is designed to investigate the impacts of HIIT and HICT combined with functional exercises and primitive reflex training on quality of life and cognitive functioning for patients with SUD. Specifically, the protocol will examine whether structured physical activity improves cognitive functioning for SUD patients relative to that of SUD patients in a control group.
2. We will examine whether different intensities of physical activity, such as HIIT/HICT versus other types of physical activity (e.g., hiking) are associated with different cognitive recovery trajectories during the subacute phase of SUD treatment.

This research protocol has several possible applications. First, it will enable future studies to reproduce the exercise protocol and increase the knowledge base on SUD and the effects of exercise. One of the more significant problems in the exercise literature is the inadequate description of exercise, such as the dose, intensity, and how exercises are implemented in studies, which limits reproducibility. This protocol and training regime would serve as a base for comparisons with other workout routines, types of exercises, and duration of exercises. Because this exercise regime will be compared with control group activities, it will be validated as having positive or negative effects for SUD patients in a subacute clinical setting.

Second, because there are so few studies on exercise and substance abuse, a protocol will make it easier to reproduce and increase the number of studies with identical exercise regimes by using this protocol as a guide. This would facilitate comparisons between studies and allow for systematic reviews and meta-analyses.

Third, if this protocol leads to an effective intervention which improves the participants’ cognitive functioning and quality of life, the training regime would be inexpensive and easy to implement in a clinical setting, as there is no need for specialized equipment or extensive staff training.

## Materials and Equipment

### Design

The design of this study is a quasi-experimental, longitudinal clinical trial, with pretest and multiple posttests. The control and intervention groups will be run sequentially, with patients naturally randomly assigned to substance abuse treatment (see below). This study will be conducted in a clinical setting as part of the daily treatment interventions conducted at the Salvation Army Treatment Center, Stavanger (FAB), including testing the efficacy of HIIT for the recovery of cognitive functioning in patients with SUD. After completion of the clinical trial, patients will be followed prospectively for 1 year.

### Control Group

This study will have an active control group of SUD inpatients recruited from the same treatment facility as the patients in the intervention group. The control group will receive treatment as usual (TAU) for SUD patients. The treatment focuses on activities of daily living (ADL), including personal hygiene (e.g., washing oneself and brushing teeth); sleep hygiene, which focuses on regular sleep and wake-up times to help normalize sleep patterns; learning or relearning how to prepare and cook food; eating in a social setting at fixed times; house cleaning and work assignments relevant to making the ward run smoothly; structured socializing and group sessions, twice a day; and structured physical activity in the form of 30–120-min daily hikes and a longer weekly hike for a duration of 3–5 h.

### Intervention Group

Participants in the intervention group will receive the same TAU as the control group, and they will also do four additional 30-min exercise protocols each week.

### Randomization

Although we will not be conducting a true randomized control trial, the control and intervention groups will be run consecutively, and random assignment to these groups will occur naturally because the external allocation system of three separate units allocates patients with different needs to the SUD treatment waiting list independently of each other. Natural allocation will be aided further by SUD patients dropping out of the waiting list for entry to treatment.

### Ethics Statement

This research project was approved by the Regional Ethics Committee for Medical Research Ethics, Western Norway (2011/1877). Written consent is collected from all participants.

All sensitive data (digital and non-digital) generated are confidential and will be treated according to the standards set by the Norwegian Data Inspectorate (Datatilsynet) and in compliance with the Health Research Act and the Personal Data Act.

### Equipment

Location: Salvation Army Treatment Center Stavanger, Rogaland, Norway.

Watch (Polar M200, M360), weight scale, stopwatch, chair, running track (Stavanger Stadium), exercise sling, two gym mats, computer for neurocognitive testing, and a timer (Tabata).

## Stepwise Procedures

Recruitment and eligibility. Patients will be recruited from the Helse Vest catchment area via the subacute treatment facility FAB. The Helse Vest catchment area consist of western Norway. FAB is mainly recruiting form the county of Rogaland and the main cities in the region Stavanger, Sandnes, and Haugesund. The patient target group for FAB is refered patients from the specialized health care system, with an addiction so serious that inpatient treatment is deemed necessary by a evaluation center. Comorbidity of light psychiatric diagnoses are accepted. We included patients of 18 years of age and upward. The patients often receive social benefits or disability pension. Education among the patient group is mixed from elementary school to university educated. We included patients mixed between first time rehabilitation to varying number of previous admission. Eligibility: SUD diagnosis, admitted as an inpatient to FAB; ≥18 years of age; passes the physical evaluation for the institution to perform intensive training; no medical history or illness that precludes participation in physical activity; and psychiatric diagnoses no more serious than the grading of light to moderate. Patients will be excluded from the study if their medical history (e.g., physical disability, disease, or injury) could interfere with or be exacerbated by physical activity. For example, patients with paralysis, severe pain, obstructive disease, glaucoma, or who are unable to sit, stand, and/or walk will be excluded, as well as patients with a severe cognitive deficit, such as dementia. A severe cognitive deficiency will be assessed based on the patients’ responses to two questions: “Do you have trouble with your memory that affects your daily life?” and “Have you been given a medical diagnosed of dementia?” Protocol deviations: If participants are absent from more than 60% of the training sessions. They are considered protocol violators. If they are not able to participate at allocated test times and complete the tests, they are considered protocol violators. They are still included in the study and tested as soon as possible. Statistical analyses will be performed both based on the principle of “intention to treat” including everyone regardless of protocol violation and “per protocol” analyses will be performed, comparing only those that have completed the protocol.

**FIGURE 1:**
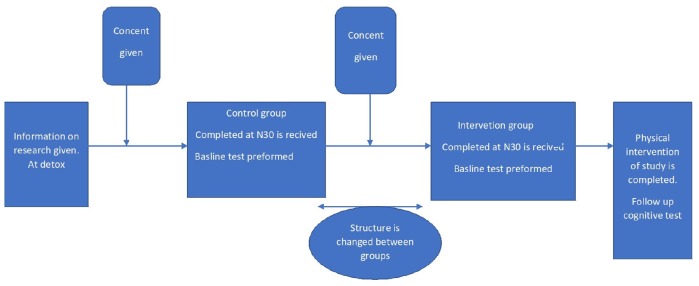
FAB, Stavanger 2019.

1. Baseline testing. The physical and neuropsychological testing will be conducted within 2 weeks of admittance to the Salvation Army Treatment Center.
2. The researchers will not conduct randomization, as there is natural allocation as a result of the SUD treatment allocation system, which will be continuous throughout the study.
3. The control group is completed first. Structure is changed in the clinic and intervention group is started up after the last control patient has left the clinic.
4. The intervention will consist of the physical exercise protocol, which will be conducted for 4 weeks.
5. The final tests of physical function will be conducted the week after the exercise protocol is completed. The prospective follow-up will consist of cognitive testing carried out at 3, 6, 9, and 12 months after the baseline test.
6. Discontinuation: Patients that don’t follow the protocol or don’t follow the TAU of the Facility will be discontinued. If they don’t complete the follow up test with in the allocated time line or neglect to perform the test they will be discontinued (Figure 1).

### Testing Procedures

#### Rockport 1-Mile Walk Test

This will be performed at the Stavanger outdoor stadium. Patients will be timed while walking 4 × 400 m, and pulse rate will be recorded with the final crossing of the finish line (George et al., 1998).

#### 30-Second Chair Stand Test

This will be conducted by having the patient perform as many sit–stand–sit cycles as possible during a 30-s test (Macfarlane et al., 2006; Millor et al., 2013), with the number of cycles completed recorded. This test will be conducted at the Salvation Army Treatment Center.

#### 1-Minute Burpee Test

This is a functional test and outcome measure. The burpee is a total body exercise used in strength training, high-intensity training, and aerobic exercise. It is designed to develop strength, agility, coordination, and aerobic performance. The test starts with the subjects in a standing position with their feet shoulder-width apart. They then drop into a squat position with their feet underneath them and their hands on the ground and quickly extend their feet in one motion to assume the front plank position with legs completely extended and back straight. Finally, they return to the squat position and then jump straight into the air as high as possible. Repeat. This entire exercise is intended to be performed in a fluid, rapid movement (Gist et al., 2014).

### Physical Exercise/Intervention Protocol

The study uses HIIT/HICT consisting of functional exercise movements combined with primitive reflex training exercises conducted in a 4-week program to examine the effects of this protocol on the recovery of cognitive function after chronic SUD. In the current literature, high-intensity training is considered a high-yield training form and usually includes three types of exercise (Unhjem et al., 2016). The best evidence indicates that a minimum of three 30–60-min sessions are needed per week (Milanović et al., 2015; Eddolls et al., 2017), over the course of 4–9 weeks of training. Because our patient population has relatively short-term stays in the facility, we set the control and intervention periods as 4 weeks each. As noted, the physical exercise protocol for the intervention group consists of four 30-min sessions per week, with an active warm-up included in each session. Each of the 30-min exercise sessions will be the same, with a target heart rate of 70–90% of the patient’s maximum heart rate (220 beats per minute minus the patient’s age). Each training session consists of a warm-up period at 60–70% of maximum heart rate (114–133 bpm), followed by exercise performed at 70–90% of maximum heart rate (134–170 bpm), with a cutoff at 90% (171 bpm), and then an active cooldown period. Polar heart rate zones will be used to control the exercise intensity throughout the training sessions. Because of the low cost and limited need for equipment, we plan to use body weight functional exercises in this study. The exercises for each training session will be performed at nine stations, with 45 s of work at each station and 15 s to rest and change stations. Participants will complete three circuits of the nine stations, with 25 s of rest between circuits. The active cooldown consists of walking for 5 min. The nine exercises are: air squats; cat/camel; reverse rowing with a sling; starfish; burpees; frog sit-ups/CrossFit sit-ups; crawling [asymmetrical tonic neck reflex (ATNR) exercise]; jumping jacks; and push-ups. Supplementary Appendix A contains further description and information about the exercises in the protocol.

### Neuropsychological Testing

The present study is an intervention study as a follow up study from the Stayer longitudinal cohort study, and we will be using the some of the cognitive tests, that the Stayer study uses to follow the mental health of their participants. The test is listed below. All the neuropsychological testing will be performed by a research assistant who is specially trained to administer neuropsychological tests. The following paragraphs summarize the cognitive tests.

#### Clinical Self-Rating Questionnaires

The Symptom Checklist-90-Revised (SCL-90-R) is a 90-item self-report symptom inventory developed by Derogatis (Derogatis and Unger, 2010) in the mid-1970s to measure psychological symptoms and psychological distress. It is designed for use with individuals from the community, as well as with individuals with either medical or psychiatric conditions. The SCL-90-R assesses psychological distress along nine primary symptom dimensions and three summary scores, which are referred to as global scores. The principal symptom dimensions are labeled Somatization (SOM), Obsessive-Compulsive (OBS), Interpersonal Sensitivity (INT), Depression (DEP), Anxiety (ANX), Hostility (HOS), Phobic Anxiety (PHOB), Paranoid Ideation (PAR), and Psychoticism (PSY). The three global measures are the Global Severity Index (GSI), the Positive Symptom Distress Index (PSDI), and the Positive Symptom Total (PST) (Derogatis and Unger, 2010).

The Satisfaction with Life Scale (SWLS) assesses subjective well-being by focusing on global life satisfaction. It does not tap related constructs such as positive affect or loneliness. The SWLS has favorable psychometric properties, including high internal consistency and high temporal reliability. Scores on the SWLS correlate moderately to highly with other measures of subjective well-being and correlate predictably with specific personality characteristics (Diener et al., 1985; Pavot et al., 1991).

The Behavior Rating Inventory of Executive Function–Adult Version (BRIEF-A) is a self-report form designed to be completed by adults 18–90 years of age, including adults with a wide variety of developmental, systemic, neurological, and psychiatric disorders, such as attention disorders, learning disabilities, autism spectrum disorders, traumatic brain injury, multiple sclerosis, depression, mild cognitive impairment, dementia, and schizophrenia. The BRIEF-A is composed of 75 items in nine non-overlapping and theoretically and empirically derived clinical scales that measure various aspects of executive functioning (Gioia et al., 2000; Kessler et al., 2005).

The Adult ADHD Self-Report Scale (ASRS) include 18 questions about the frequency of recent the Diagnostic and Statistical Manual of Mental Disorders (DSM-IV) Criterion A symptoms of adult ADHD. The ASRS screener includes six out of these 18 questions that were selected based on stepwise logistic regression to optimize concordance with the clinical classification. ASRS responses were compared to blind clinical ratings of DSM-IV adult ADHD in a sample of 154 respondents who previously participated in the US National Comorbidity Survey Replication (NCS-R), oversampling those who reported childhood ADHD and adult persistence (Reitan and Wolfson, 2004).

The Alcohol Use Disorders Identification Test (AUDIT) consists of a 10-item Core questionnaire and an 8-item Clinical procedure. AUDIT was designed to identify hazardous drinkers (whose drinking increases their risk of alcohol-related problems, although alcohol-associated harm has not yet occurred); harmful drinkers (who have had recent physical or mental problems related to their drinking, but who are not alcohol-dependent); and people with alcohol dependence (Tombaugh, 2004).

The Drug Use Disorders Identification Test (DUDIT) is an 11-item self-report questionnaire developed to screen individuals for drug problems (Gioia et al., 2000).

The quality register is a national semi-structured interview for the Norwegian healthcare system that is used as a tool for patient feedback and the mapping of patient health, quality of life, employment, housing, and education.

#### Neuropsychological Tests

The Montreal Cognitive Assessment (MoCA) (Voluse et al., 2012) takes approximately 10 min to administer and is designed to detect mild cognitive impairment in elderly participants who score in the normal range on the Mini-Mental Status Examination. The MoCA uses 30 items to assess multiple cognitive domains, including: short-term memory (5 points); visuospatial abilities via clock drawing (3 points), and a cube copying task (1 point); executive functioning via an adaptation of the Trail Making Test Part B (1 point), phonemic fluency (1 point), and verbal abstraction (2 points); attention, concentration, and working memory via target detection (1 point), serial subtraction (3 points), digits forward (1 point), and digits backward (1 point); language via confrontation naming with low-familiarity animals (3 points) and repetition of complex sentences (2 points); and orientation to time and place (6 points) (Voluse et al., 2012). The MoCA is scored by obtaining an item total, and the authors recommend a clinical cutoff score of 26 (Voluse et al., 2012).

The Stroop Color and Word Test (SCWT) is used extensively to assess the ability to control the cognitive interference that occurs when the processing of a specific stimulus feature impedes the simultaneous processing of a second stimulus attribute, which is well known as the Stroop effect. The Stroop test has been used in previous trials on physical exercise and cognition and is a sensitive measure of prefrontally mediated cognitive functions (Bohn et al., 1995; Nasreddine et al., 2005).

The Trail Making Test is a test of visual attention and task switching. It consists of two parts in which the subject must connect a set of 25 dots as quickly and accurately as possible (Golden and Freshwater, 1978). The test provides information about visual search speed, scanning, speed of processing, and mental flexibility, as well as executive functioning (Arnett and Labovitz, 1995; Arbuthnott and Frank, 2000; Martínez et al., 2016; Scarpina and Tagini, 2017).

## Anticipated Results

In this study, we expect the participants in both the control and intervention groups to show progress in physical and mental health after a period of substance abuse. We believe that the data generated in this project will mostly be at the interval or ratio level (whether the rating of perceived exertion is ordinal or interval data will not be discussed here). We will use the mean as a measure of central tendency for the group results for the neurocognitive test, while the reflexes we use frequency and proportions, and the standard deviation to express the spread of results within the group. If useful, the standard error of the mean (SEM) will be used to express the accuracy of the mean value. If the results are highly skewed (skewness > 1.5), we will use a log transformation and geometric mean together with a 95% confidence interval to express central and spread tendencies. To compare results over time from pretest to posttests, and between-group differences, we will use repeated measures ANOVA/*t*-tests with Sidak/Holm *post hoc* tests, where we have collected three or more measurements a linear mixed modeling accounting for the missing data will be used. Group comparisons will be made with an ANOVA/*t*-test for independent groups. If the test of normality should fail (Kolmogorov–Smirnov with Lilliefors correction), the corresponding non-parametric tests will be used, i.e., the Wilcoxon–Mann–Whitney rank sum test for independent *t*-tests and repeated measures ANOVA for ranks (Tukey’s *post hoc* test). Statistical significance will be set at a *p*-value of ≤0.05, and effect sizes will be calculated according to Cohen (1988), with the help of an online effect size calculator (the CEM effect size calculator, Center for Evaluation and Monitoring, Durham University, United Kingdom^1^). An effect size of ≥0.5 will be considered clinically relevant (two-sided). If there is a need for building indexes of different types of scores, *Z*-scores will be used. Based on previously published data on fitness development and depression scores, we should be able to attain statistical power of 0.8 with at least 10 subjects in each of the two groups. Nevertheless, we have set target recruitment at *n* = 30 for each group.

## Discussion

This study is the first of its kind where investigations of changes in cognitive function as a direct effect of structured physical activity are assessed when the physical exercises are based on bodyweight exercises and functional movement structed into a high-intensity circuit. We hypothesize that the benefits of doing it this way will kick start the body’s own regeneration process and brain plasticity to repair the damage that has occurred do to drug use due to release of signaling substances such as brain-derived neurotropic factor. In conducting this exercise program, we observe changes in both groups.

### Limitations of the Study

Because the intervention and control groups will be run consecutively rather than concurrently, there is the potential for non-equivalent time periods to create bias. However, we chose this approach because previous studies had suffered recruitment failures and high dropout rates. The length of the proposed study is not fixed, and because of our funding and support, this study can continue until the quota for participants is met.

This study is designed to accommodate clinical realities. Thus, different SUD groups are not fractionated during treatment, and the project protocol does not divide different groups of SUD after clinical classification. This may create some problems with the results from baseline because of various issues with the abuse of different drugs and possible side effects that might affect the test results differently. We will account for this Statistically.

Finally, the patient population will be recruited straight out of a detoxification clinic, which will most likely influence the test results. However, this may actually be an advantage, because having access to patients so quickly after controlled, medically supervised detoxification may give us a more accurate picture of their mental status when the patients are at their lowest point in their recovery trajectories.

The Norwegian Ministry of Health and Social Affairs and the Salvation Army of Norway have provided financial support for this study.

## Ethics Statement

The studies involving human participants were reviewed and approved by the Regionale komiteer for medisinsk og helsefaglig forskningsetikk (2011/1877/REK vest). The patients/participants provided their written informed consent to participate in this study.

## Author Contributions

All authors conceived the study. ØA wrote the manuscript and contributed with scientific/clinical expertise. EF and KB also contributed to writing this manuscript. SN contributed to project oversight and resource control. A-LN contributed to designing the protocol and retrieval of data.

## Conflict of Interest

The authors declare that the research was conducted in the absence of any commercial or financial relationships that could be construed as a potential conflict of interest.

## Footnotes

1 http://www.cemcentre.org/evidence-based-education/effect-size-calculator
