# Supplementary material for: The Effect of High-Intensity Interval/Circuit Training on Cognitive Functioning and Quality of Life During Recovery From Substance Abuse Disorder. A Study Protocol
Source: Front Psychol. 2019 Nov 15;10:2564. doi: 10.3389/fpsyg.2019.02564 (PMC6873325; doi:10.3389/fpsyg.2019.02564)
Supplement: Supplementary file 1 [file Data_Sheet_1.PDF]

Appendix A

The physical exercise protocol consists of 30-minute sessions with active warm-up included in the sessions four times a week with a target heart rate of 70-90%. Each training session is identical and divided in to with warm-up done at 60-70% 114-133bpm, work is conducted at 70-90% 134-170 bpm. With a cut off at 90% 171 bpm and active cool down. Using the polar heart rate zones to control the exercise intensity. If no watches are available, the Borge scale can be used to monitor the intensity of the workout. The exercise is done in 9 stations of 45 seconds work. Fifteen seconds rest and change of station for three circles with 25 seconds rest between each circle. The training session consisting of nine individual exercises:

1. Air squats
2. Cat/Camel
3. Inverted rowing with sling/TRX
4. Stare fish,
5. Burpee,
6. Sit-ups with frog legs,/CrossFit sit-ups
7. Crawl (ATNR)
8. Jumping jacks
9. Push up

The exercises mentioned above can be peeled-back to accommodate different body shapes, fitness level and disabilities. Examples of modifications for each exercise added as an extra point for each activity.

Air squats:

The Air squats help to build both a solid strength foundation and balance in your lower body. They target your thighs, hamstrings, quadriceps, and glutes, in particular, helping you to add muscle mass in these areas. Because balance is necessary, air squats can also engage your core.

Start with standing with your feet hip-width apart with your toes pointed slightly outward. Your arms should be hanging loosely by your side. Then engage your core muscles and push out your chest somewhat by pulling your shoulder blades towards each other. Bend your knees and squat down as if you were sitting into a chair. Keep your weight on your heels and keep your core tight. Your eventual goal will be to touch your glutes to the back of your calves, but if you can only get to parallel right now, that’s fine. Make an effort to keep your knees externally rotated (don’t let them collapse inward). As you lower down, you can either raise your arms straight in front of you or keep them bent in front of your chest. Focus on keeping your torso upright and core tight. Straighten your legs and squeeze your butt to come back up, lowering your arms back to your side.

Peel-back: use of support, e.g. wall or rope. So that one can take off some of the body weight and use arms to help in the movement,

Cat/Camel

Get onto your hands and knees, with your knees spaced hip-width apart and your hands directly beneath your shoulders. Tighten your abdominal muscles and arch your spine upward toward the ceiling. Hold the position for at least 10 seconds, then slowly relax your back. Allow your stomach to fall toward the floor, bring your shoulders together and stretch your back downwards into a swayback position. Hold for 10 seconds, then return to the starting position. Keep the muscles in your sides, abdominals and lower back tight throughout the entire exercise to help stabilize your spine and keep your back in a neutral position. To prevent injury, perform the cat and camel exercise slowly and gently. Nerve force yourself to stretch to the point of pain. Slow your pace, reduce the number of repetitions or take a break if you experience any pain or extreme discomfort while exercising.

Peel-back is not needed for this exercise. It's performed in the range that is comfortable and feels normal for the patient.

Inverted rowing with a sling/TRX or Olympic rings

Grab the handlebars/rings of the sling. Grip it in a way that is most natural for the person doing the exercise. Walk backwards away from the sling. Lean backwards, so arms are extended. Contract the abs for core engagement and try to keep the body in a straight line. How hard/ heavy the exercise is regulated by walking forward at the same time as arms are extended and sling is loaded. Exercise is performed by pulling body toward sling handles/rings until chest touches the handles.

Alternative exercise is Inverted bodyweight row using a bar.

- Lie on the floor underneath the bar (which should be set just above where you can reach from the ground).
- Grab the bar with an overhand grip (palms facing AWAY from you).
- Contract your abs and try to keep your body a completely straight line. Your ears, shoulders, hips, legs, and feet should all be in a straight line.
- Pull yourself up to the bar until your chest touches the bar.
- Lower yourself back down**.**

Peel-back: is regulated with walking more upright to reduces stress (difficulty of exercise) or walking forward, and becoming more parallel with the floor and the body is more horizontal to increase movement.

Starfish

Lie back on a bean bag, bench. Tilt head back, arms up and out, legs wide. Bring arms in and crossed, right over left. Bring legs in at the same time, right over left. While breathing in, to the count of 5: Bring arms back out. Bring legs back out. Bring arms in and crossed, left over right. Bring legs in at the same time, left over right

Peel back: For this activity, there no specific peel back. It’s about being as close to the intended original movement in pain-free movement. If there is need one can reduce the range of the movement in the exercise to accommodate restricted movement.

Burpee

The Burpee is a whole body exercise used in strength training, high-intensity training and aerobic exercise. It is designed to develop strength, agility, coordination and aerobic performance.
Start in a standing position with your feet shoulder-width apart. Drop into a squat position with your feet underneath you and your hands on the ground. Quickly extend your feet in one motion to assume the front plank position with your legs completely extended and your back straight. Return to the squat position then jump straight into the air as high as possible. Repeat. This entire exercise is intended to be performed in a fluid, rapid movement

Peel-back: do not need to be in plank position, can drop all the way and lay flat on the floor. Can walk up instead of jumping up in one movement.

Sit-ups

Sit on the floor with the soles of the shoes touching, legs butterflied out to the side - Take a towel, pillow, sweater or AbMat and place it on the ground behind the bottom Execution - Sit back so the object fills the void of the lower back with the ground - Bring the arms overhead while lowering to the ground and touch the floor behind - Using the arms, sit up and touch the feet in front so the torso is at a 90 degree angle to the hip.

Peel-back: Stopping the movement so that the core is engaged if the pain is induced doing the full range movement.

Lizard Crawl

Crawling exercises are multi-joint movements that engage the entire body while emphasizing the core and shoulder muscles Start by lying face down. Turn head to the left. As the head is rotating, slide elbow down toward waist and bend left knee up towards waist. Eyes are locked to the thumb and follows its path throughout the exercise.

Peel-back: the exercise is performed as good as one can towards the original. Reduce movement if restricted or pain occurs.

Jumping jacks

The heart rate is increased while performing jumping jacks, which increases the amount of blood circulation containing nutrients and oxygen to the muscles. Jumping jacks' aids in improving the stamina of the body and its overall endurance level. You need to stand straight with your feet together and arms by your sides. Quickly raise your arms overhead while simultaneously jumping on your feet on either side. Make an inverted V shape with your legs with every jump. To avoid any injuries, make sure you land gently. Always wear good shoes while doing an exercise like jumping jacks.

Peel-back: One for can be stationary while the other jumps in and out, e.g. mentioned above. Or no jumping if there is knee pain, the exercise is conducted walking sideways instead.

Push up

The Push-Up is a callisthenic or body-weight exercise designed to develop the chest, triceps, shoulders and core. Start by supporting your body on the balls of your feet and your palms, positioning the later slightly wider than shoulder-width apart. Straighten your arms without locking your elbows. Lower your torso while keeping your back and buttocks flat, until your chest is just a fraction of an inch from the floor. Push yourself back to the starting position.

Peel-back: push up can be performed on knees, change of hands positions compared to shoulder to make it easier. Use of a box or bench to elevate the upper body higher than the lower body to make the exercise easier.

Resources:

9 Essential Crossfit Movements You Need To Know. (n.d.). Retrieved from http://www.crossfitedify.com/blog/2019/4/11/9-essential-crossfit-movements-to-he

Push Ups - Body-solid Fitness. (n.d.). Retrieved from https://www.bodysolid.com/exercises/article/4/push_ups

7 Health Benefits Of Jumping Jacks You Never Knew. (n.d.). Retrieved from https://doctor.ndtv.com/living-healthy/jumping-jack-health-benefits-why-jumping

Reebok Crossfit One Movement Demo "abmat Situp" - Youtube. (n.d.). Retrieved from https://www.youtube.com/watch?v=EhG_x1bLHwE

Burpees - Body-solid Fitness. (n.d.). Retrieved from https://www.bodysolid.com/exercises/article/69/burpees

: Cat And Camel Back Exercise | Chron.com. (n.d.). Retrieved from https://livehealthy.chron.com/cat-camel-back-exercise-7452.html

How To Properly Perform Inverted Row Tutorial - Youtube. (n.d.). Retrieved from https://www.youtube.com/watch?v=XkqFYy1M7oM

Musall, Lisa - Ot / Reflex Integration Exercises. (n.d.). Retrieved from https://www.cattlv.wnyric.org/Page/2427
